# Polarization properties and Umov effect of human hair

**DOI:** 10.1038/s41598-023-50457-x

**Published:** 2024-01-03

**Authors:** Alaa Hamdoh, Sawyer Miller, Yufei Gao, Yang Zou, Matthew Smith, Linan Jiang, Stanley Pau

**Affiliations:** 1 Electrical and Computer Engineering, University of Arizona, Tucson, AZ 85721 USA; 2 James C. Wyant College of Optical Science, University of Arizona, Tucson, AZ 85721 USA; 3 Axometrics, Inc., Huntsville, AL 35806 USA

**Keywords:** Biotechnology, Optics and photonics

## Abstract

This study delves into the polarization properties of various hair colors using several techniques, including polarization ray tracing, full Stokes, and Mueller matrix imaging. Our analysis involved studying hair in both indoor and outdoor settings under varying lighting conditions. Our results demonstrate a strong correlation between hair color and the degree of linear polarization. Specifically, light-colored hair, such as white and blond, exhibits high albedo and low DoLP. In contrast, dark hair, like black and brown hair, has low albedo and high DoLP. Our research also revealed that a single hair strand displays high diattenuation near specular reflections but high depolarization in areas with diffuse reflections. Additionally, we investigated the wavelength dependency of the polarization properties by comparing the Mueller matrix under illumination at 450 nm and 589 nm. Our investigation demonstrates the impact of hair shade and color on polarization properties and the Umov effect.

## Introduction

Human hair appearance plays a crucial role in recognizing and perceiving a person’s status, age, and health. Hair can look different depending on its type, color, shape, and treatments such as coloring and perming. While existing studies have explored how light reflects, refracts, scatters, and attenuates by hair^1–5^, they often overlook the effect of light polarization. Past studies of the optical properties of hair include measurements of attenuation, luster, and scattering^4,6–9^ and their relationship to melanoma development^5^. By considering polarization, we can gain additional information and understanding of the optical properties of hair. Depending on the illumination and observation condition, the albedo of hair can be very different when illuminated by unpolarized light or polarized skylight or when observed using polarized sunglasses.

**Figure 1:**
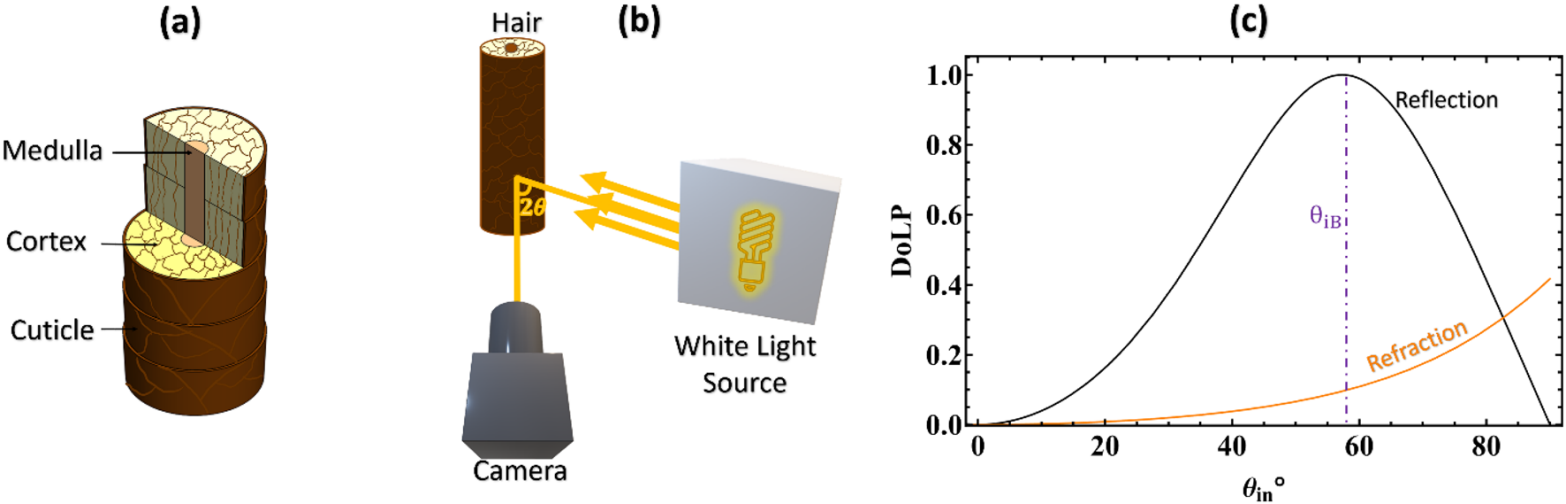
(**a**) Schematic diagram shows the cross-section and three component layers of a hair strand. (**b**) Schematic diagram shows the indoor experimental setup for full Stokes imaging. (**c**) The DoLP of a dielectric surface of hair is plotted as a function of angle of incidence for both reflected and refracted light. Vertical line marks the Brewster angle ${\theta }_{iB}$.

An average strand of hair measures approximately 60 to 100 µm in diameter, forming a cylindrical shape with an approximately circular cross-section^10^. It comprises three distinct layers of keratin material: the medulla, cortex, and cuticle, as illustrated in Fig. 1a. Typically, the melanin granules in the inner cortex determine hair color^11^. Both internal and external structures of hair affect the amount of light scattering. Hair does have birefringence^12^ due to keratin, which is its main component and can be semi-crystalline. Hair has an estimated birefringence $\Delta n$ of 1–0.5% of its refractive index^12^ and an average refractive index of 1.54 to 1.56 in the visible spectrum^13^.

In this work, we study the optical properties and albedo of hairs in different colors using full Stokes and Mueller matrix imaging techniques. The polarization properties of single strands of hair and hair bundles are measured under different types of illumination in indoor and outdoor environments. We examine the Umov effect in the hair, which relates the albedo of an object to the degree of linear polarization (DoLP) of the reflected light. Polarization imaging can separate specular and diffuse reflection^14,15^ in addition to reflected and transmitted light^16^. In addition to color imaging, polarization imaging can provide additional information about different hair types, as each hair type has unique signatures of diattenuation, retardance, and depolarization. This study has applications in hair identification and characterization, cosmetics, medical diagnosis, computer graphics, and laser therapy. The analysis and techniques utilized in this work can also be applied to studying other types of fibers, including natural fiber, such as silk, and synthetic fiber, such as polyester.

## Theory

When a dielectric material such as hair is illuminated by unpolarized light, the reflected and refracted light are polarized. The degree of linear polarization (DoLP) describes the polarization state of light with a value ranging from zero to one. DoLP is equal to one for completely linearly polarized light and zero for completely unpolarized light.

We introduce the Stokes parameters to describe the polarization state of light in more detail. These are derived from light intensity measurements in different polarization states and are related to the Fresnel coefficients, which describe how light is reflected and transmitted at an interface. We denote here the Stokes vector $\overrightarrow{S}$ with the four components: ${S}_{0}$ is the intensity sum between the horizontal and vertical linear polarization, ${S}_{1}$ is the intensity difference between the horizontal and vertical linear polarized light, ${S}_{2}$ is the intensity difference between the 45° and 135° linear polarized light, and ${S}_{3}$ is the intensity difference between the right-circular and left-circular polarized light.

With the foundational understanding of Stokes parameters, we can now express them specifically in terms of the Fresnel reflection and transmission coefficients. When unpolarized light is incident on a dielectric surface, the unpolarized light is converted to polarized light after reflection and refraction. For reflected light, the Stokes parameters ${\overrightarrow{S}}_{R}$ are given by^17^:

1a $${S}_{0R}=cos{\theta }_{i}\left({R}_{s}{R}_{s}^{*}+{R}_{p}{R}_{p}^{*}\right),$$

1b $${S}_{1R}=cos{\theta }_{i}\left({R}_{s}{R}_{s}^{*}-{R}_{p}{R}_{p}^{*}\right),$$

1c $${S}_{2R}=cos{\theta }_{i}\left({R}_{s}{R}_{p}^{*}+{R}_{p}{R}_{s}^{*}\right),$$

1d $${S}_{3R}=i\,cos{\theta }_{i}\left({R}_{s}{R}_{p}^{*}-{R}_{p}{R}_{s}^{*}\right),$$

where the factor $i$ in (1d) is $\sqrt{-1}$. These equations incorporate the cosine of the incidence angle, reflecting the reflection coefficients’ angular dependency.

The reflection coefficients are^17^:

2 $${R}_{s}=\frac{{sin}^{2}({\theta }_{i}-{\theta }_{t})}{{sin}^{2}({\theta }_{i}+{\theta }_{t})},$$

3 $${R}_{p}=\frac{{tan}^{2}({\theta }_{i}-{\theta }_{t})}{{tan}^{2}({\theta }_{i}+{\theta }_{t})}.$$

For refracted or transmitted light, the Stokes parameters ${\overrightarrow{S}}_{T}$ are^17^:

4a $${S}_{0T}=ncos{\theta }_{i}\left({T}_{s}{T}_{s}^{*}+{T}_{p}{T}_{p}^{*}\right),$$

4b $${S}_{1T}=ncos{\theta }_{i}\left({T}_{s}{T}_{s}^{*}-{T}_{p}{T}_{p}^{*}\right),$$

4c $${S}_{2T}=ncos{\theta }_{i}\left({T}_{s}{T}_{p}^{*}+{T}_{p}{T}_{s}^{*}\right),$$

4d $${S}_{3T}=in\,cos{\theta }_{i}\left({T}_{s}{T}_{p}^{*}-{T}_{p}{T}_{s}^{*}\right).$$

In these expressions, $n$ is the refractive index of the medium into which the light is transmitted.

The transmission coefficients are^18^:

5 $${T}_{s}=\frac{{\text{sin}}\,2{\theta }_{i}\,{\text{sin}}\,2{\theta }_{t}}{{sin}^{2}({\theta }_{i}+{\theta }_{t})},$$

6 $${T}_{p}=\frac{{\text{sin}}\,2{\theta }_{i}\,{\text{sin}}\,2{\theta }_{t}}{{sin}^{2}\left({\theta }_{i}+{\theta }_{t}\right){cos}^{2}({\theta }_{i}-{\theta }_{t})}.$$

With these parameters defined, we can calculate the DoLP, angle of linear polarization (AoLP), and degree of circular polarization (DoCP) for both reflected and transmitted light to understand how hair affects the polarization of light upon reflection and transmission^18^. In full Stokes imaging, as shown in Fig. 1b, the Stokes vector of the reflected light is measured.

7 $$DoLP=\frac{\sqrt{{S}_{1}^{2}+{S}_{2}^{2}}}{{S}_{0}},$$

8 $$AoLP=\frac{1}{2} {tan}^{-1}\left(\frac{{S}_{2}}{{S}_{1}}\right),$$

9 $$DoCP=\left(\frac{{S}_{3}}{{S}_{0}}\right).$$

Moving forward, we consider the DoLP in the context of Brewster’s angle. For a flat dielectric surface, with the light incident at Brewster’s angle at 57.3° and assuming the refractive index of ${n}_{hair}=1.56$^19^ we have:

10 $${\theta }_{iB}={tan}^{-1}\left({n}_{hair/{n}_{air}}\right).$$

As per Snell’s law

11 $${{n}_{air}\,sin\,\theta }_{iB}= {{n}_{hair}\,sin\,\theta }_{r}.$$

${n}_{air}$ is the refractive index of air. Empirical observations can be tied to these theoretical predictions. As shown in Fig. 1c, the plot of the DoLP of hair for reflected and transmitted light confirms that the DoLP of the reflected light is maximum and equal to unity at Brewster’s angle. This is consistent with our theoretical understanding that at Brewster's angle, reflected light is completely linearly polarized. The transmitted light, on the other hand, shows a monotonically increasing function of DoLP with the angle of incidence.

The Mueller matrix $M$ is a $4\times 4$ matrix representation of interaction with the Stokes vector and is used to describe the polarization properties of an optical component. Here we apply Mueller calculus to describe the polarization properties of hair, including depolarization, diattenuation, and retardance. Depolarization describes how much polarized incident light becomes unpolarized after a light-matter interaction^20,21^.

12 $$M=\left[\begin{array}{cccc}{M}_{00}& {M}_{01}& {M}_{02}& {M}_{03}\\ {M}_{10}& {M}_{11}& {M}_{12}& {M}_{13}\\ {M}_{20}& {M}_{21}& {M}_{22}& {M}_{23}\\ {M}_{30}& {M}_{31}& {M}_{32}& {M}_{33}\end{array}\right].$$

The first row of $M$ describes the diattenuation, and the first column describes the polarizance. The diattenuation varies from 1 for an ideal polarizer to 0 for an element that transmits all polarization states equally, such as an ideal optical retarder. Depolarizers scramble the state of polarization and convert polarized light into unpolarized light. Depolarization is usually associated with scattering, particularly multiple scattering. Retardance describes the phase difference between two orthogonal polarizations and can be calculated from the Mueller matrix using the Lu-Chipman decomposition^22^. In Mueller matrix imaging, the spatially varying $M$ of an object is measured. The diattenuation and depolarization are related to the coefficients of $M$.

13 $${\text{Diattenuation}}=\frac{\sqrt{{M}_{01}^{2}+{M}_{02}^{2}+{M}_{03}^{2}}}{{M}_{00}}.$$

To calculate the DI (depolarization index) for a nondepolarizing Mueller matrix M, one needs to use the denominator, which represents the radius of the hyperspherical surface^23^. The DI is determined by measuring the fractional distance of the Mueller matrix along a line segment from the DI to the hyperspherical surface of the nondepolarizing Mueller matrices. In this paper, we use the following DI definition.

14 $$\mathrm{Depolarization\,\, Index}=1-\frac{\sqrt{\left(\sum_{{\text{i}},{\text{j}}}{{{\text{M}}}_{{\text{ij}}}}^{2}\right)-{{{\text{M}}}_{00}}^{2}}}{\sqrt{3}{{\text{M}}}_{00}}.$$

The Umov effect, which oftentimes is applied to astronomical objects, states that the DoLP of scattered light is inversely related to the albedo $w$. The relationship can be applied to a reflective object and be approximated by the following equation^24^

15 $$DoLP\left(w\right)=\frac{\Delta p}{p+w},$$

where $p$ is the volume-average single-particle phase function (VSPF) that describes the angular distribution of scattered light of a group of particles and $\Delta p$ is the difference of VSPF between two orthogonal linear polarization directions. Here we treat $\Delta p$ and $p$ as empirical fitting parameters. Albedo is the proportion of the incident light that is reflected by a surface. In this work, we measure the relative albedo normalized by a peak value and not the absolute albedo.

## Methods

### Hair samples

Hairs in various colors, black, brown, blond, red, dark red, blue, dusty blue, purple, yellow, green, and white, were studied in the indoor experiments. The hair samples were Human Remy Hair purchased from Amazon under RINBOOOL.

### Hair bundle indoor and outdoor experiments

We utilized a Lucid Vision Labs Triton division of focal plane (DoFP) polarimeter, model TRI050S-PC. The polarimeter is a monochrome device, has a detector resolution of 2048 × 2448 pixels, and measures linear polarization states, i.e. ${S}_{0}$, ${S}_{1}$ and ${S}_{2}$. To measure ${S}_{3}$ and DoCP, an achromatic quarter waveplate was placed in front of the imaging lens. The quarter waveplate, which operates in the 400–900 nm range, was manufactured by Bolder Vision Optik Inc. The imaging lens was a Canon 102 mm lens set to infinity focus and f/5.6 aperture.

### Single hair experiment

The Mueller matrix images of different single hair strands were measured using an Axometrics AxoStep Mueller matrix imaging polarimeter. The imaging polarimeter has a 100X objective lens and a CMOS camera with a resolution of 1320 × 1024 pixels and a 10-bit depth. This polarimeter uses LED light sources with a full width at half maximum (FWHM) of around 10 nm at wavelengths of 450 nm and 589 nm in our experiments.

## Experiments

### Hair bundles

#### Indoor environment

Hair bundles of different colors were illuminated using an unpolarized softbox lighting kit. The light source has 140 LEDs with a color temperature equal to 5700 K.

**Figure 2:**
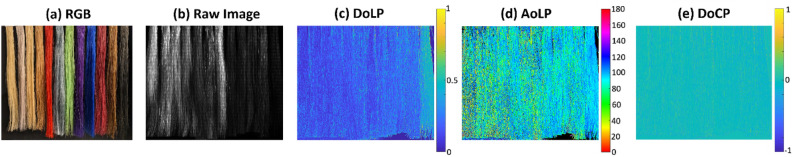
(**a**) Color image of hair bundles is shown. (**b**) An intensity image (${S}_{0}$) of hair bundles is shown. (**c**) The DoLP image of hair bundles is shown. (**d**) The AoLP image of hair bundles is shown. Black color denotes zero DoLP where AoLP is not well defined. (**e**) The DoCP image of hair bundles is shown.

In Fig. 2a, one can see a colorful image featuring twelve hair bundles of varying shades. Moving from right to left, the hair colors range from black to brown, dark red, blue, purple, light green, dusty blue, red, blond, and three different shades of white. The Stokes images of the hair bundles were measured using a polarization camera. The intensity of the hair bundles, ${S}_{0}$, is shown in Fig. 2b. As expected, the lighter hair bundles, which have less melanin and absorb less light, exhibit a higher albedo due to greater scattering.

Conversely, darker hair bundles with more melanin and higher absorption have a lower albedo. Figure 2c shows the DoLP image, with the highest DoLP value of 0.45 belonging to the black hair bundle on the far right and the lowest value below 0.2 belonging to the light green hair bundle. The high DoLP of black hair is attributed to low background diffuse light reflection and high specular linearly polarized light reflection close to and at the Brewster angle. The angle of linear polarization (AoLP) image is displayed in Fig. 2d, with the angle of specularly reflected linearly polarized light aligning closely with the direction of the hair length at around 90°. Finally, Fig. 2e shows the DoCP image, which is uniform with a value close to zero, indicating that the reflected light has no or very little elliptical or circular polarization.

**Figure 3:**
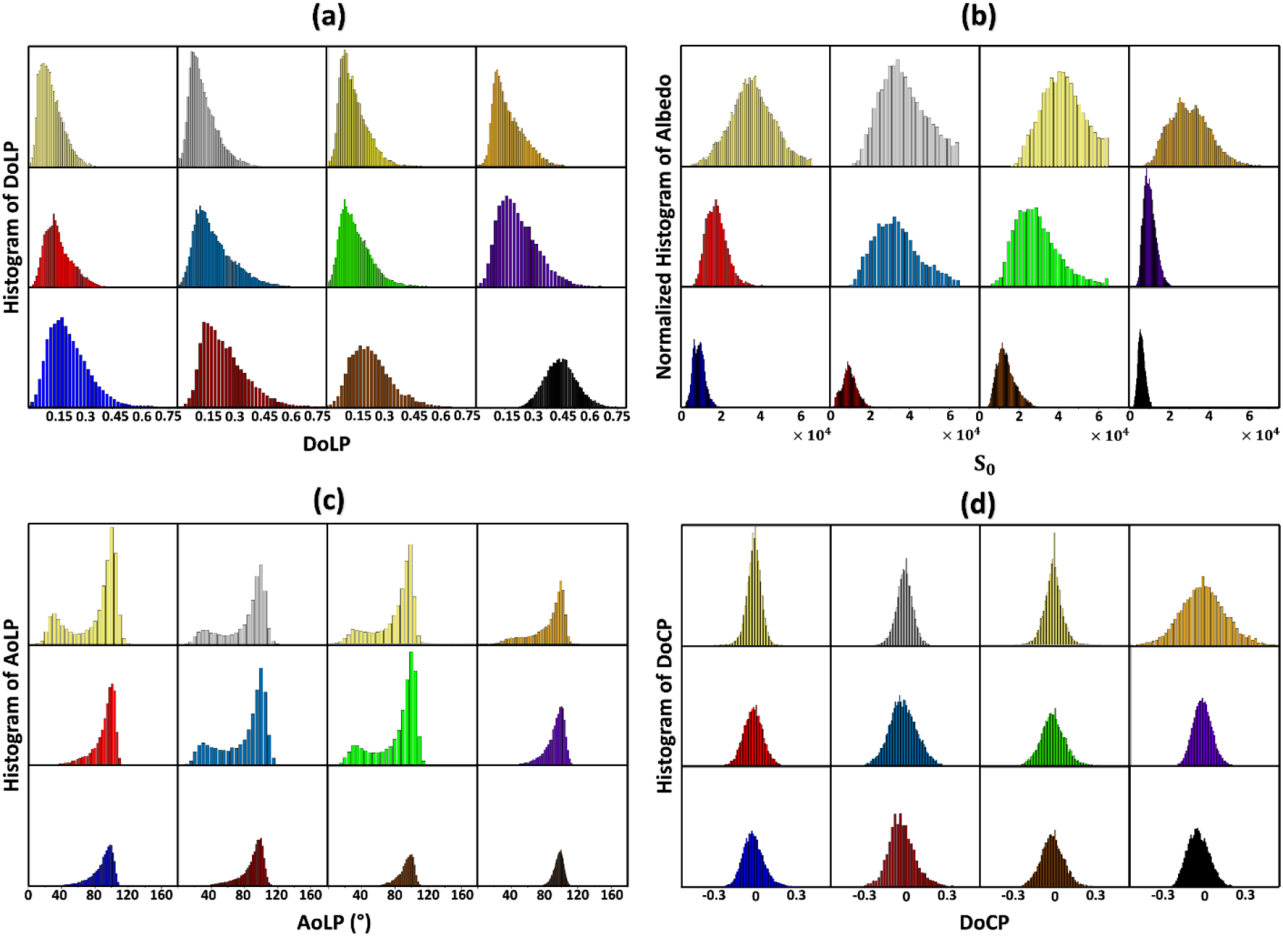
(**a**) DoLP histograms of hair bundles are shown. (**b**) Normalized albedo histograms of hair bundles are shown. (**c**) AoLP histograms of hair bundles are shown. (**d**) DoCP histograms of hair bundles are shown. For the top row, from left to right, the hair bundle colors are light yellow-white, gray-white, yellow-white, and blond. For the middle row from left to right, the hair bundle colors are red, dusty blue, green, and purple. For the bottom row, from left to right, the hair bundle colors are blue, dark red, brown, and black.

Histograms of DoLP, Albedo, AoLP, and DoCP for the hair bundles are summarized in Fig. 3. They are calculated from Stokes image of the hair bundles taken under the same illumination condition and are plotted in colors corresponding to the hair bundles. A set of DoLP histograms for the twelve hair bundles of different colors is shown in Fig. 3a. The histogram of DoLP of the black hair bundle with a symmetric bell-shaped profile has a peak around 0.45 and a maximum value of around 0.8*.* The two leftmost histograms in Fig. 3a, representing the lightest white-shade hair bundles, both present asymmetric left-skewed profiles. They also have lower DoLP values than black hair bundle: a peak around 0.1 and a maximum value of approximately 0.45. Figure 3b presents a set of normalized albedo histograms for the twelve colored hair bundles. For the black hair bundle, the histogram has a symmetric profile with a peak at approximately an albedo value of 0.7 × 10^4^ and a maximum albedo value of approximately 1.55 × 10^4^. Conversely, the histogram of the normalized albedo of the whitest hair bundle shows an asymmetric profile of reduced albedo. It has a peak albedo value of 4 × 10^4^ and a maximum albedo value of about 6.5 × 10^4^. Figure 3c displays a collection of AoLP histograms representing the twelve colored-hair bundles attributed to the curved surface of the hair. The ratio of the areas of the two peaks provides an estimate of the ratio between specular and diffuse reflections, which is generally smaller for light hair bundles compared with dark hair bundles. This result supports the observation that the scattering coefficient of lightly pigmented hair is approximately four to five times higher than that of black hair^4^. Figure 3d displays a group of DoCP histograms of the colored hair bundles. Notably, the histograms for all hair bundles of various colors remain relatively consistent; each has a bell-shaped profile with a DoCP peak value close to zero.

**Figure 4:**
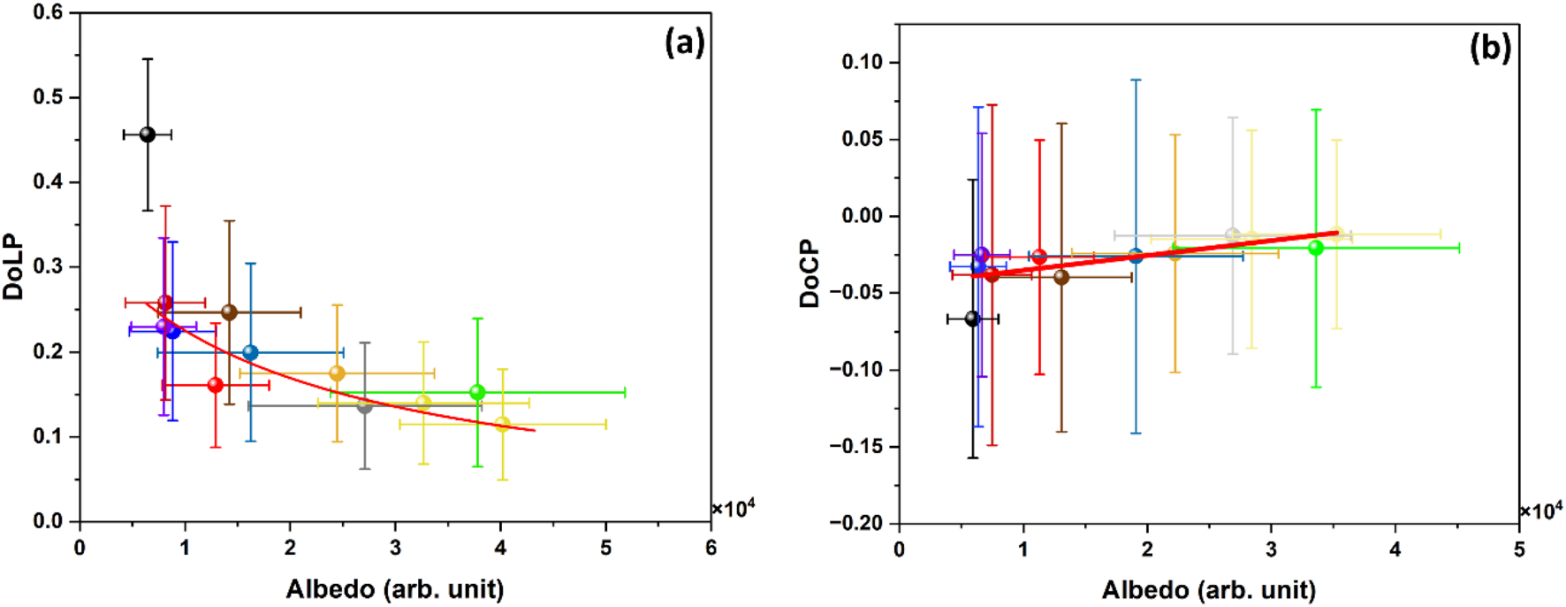
(**a**) Scatter plot of DoLP and albedo of twelve hair colors is shown. Error bars represent the standard deviation of the histogram for DoLP and albedo. The solid line is the least square fit to an equation of the Umov effect ($\Delta p=6545$ and $p=15919$), and R^2^ = 0.54. (**b**) Scatter plot of DoCP and albedo of twelve hair colors is shown. Error bars represent the standard deviation of the histogram for the DoCP and albedo. The solid line is the least square fit to a line. The color of each point is chosen to match the color of the hair bundle.

The albedo of human hair and the DoLP of polarized light reflecting from it can be described by the Umov effect, as shown in Fig. 4a for different hair bundles under broadband unpolarized white light illumination. The darker hair bundles have large DoLP values but low albedos, while the lighter hair bundles have high albedos but low DoLP values. The largest DoLP, approximately 0.45, is found in the black hair bundle, while the lowest DoLP, approximately 0.1, is in the white hair bundle. The measured data follows the inverse relationship between the DoLP and albedo of the Umov effect. As a comparison, Fig. 4b shows the DoCP and albedo under broadband unpolarized white light illumination. In this case, there is little variation in the DoCP among different colored hair bundles with different albedos, which are close to zero. Elliptical and circular polarizations are rarely seen in nature^25^ because they are generated by second and higher-order scattering of unpolarized light. The DoCP calculated based on the polarized ray tracing method is discussed in the Supplementary Discussion, and the results are consistent with the outcome of the experiments.

#### Outdoor environment

Light from the sun becomes partially linearly polarized through atmospheric scattering, with the maximum theoretical DoLP estimated to be around 0.8^14,26^. In this section, we study the polarization state of light scattered from hair under partially polarized outdoor illumination.

**Figure 5:**
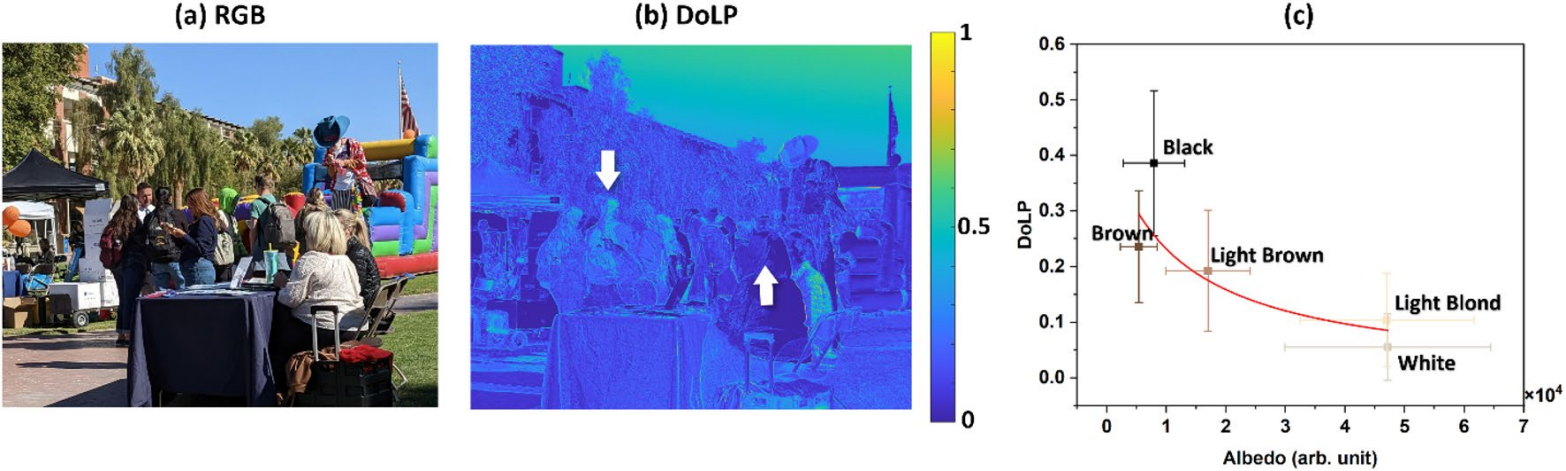
(**a**) A color image shows people with different hair colors outside the student union at the University of Arizona on a sunny day. (**b**) The DoLP image of the same outdoor scene is shown. White-colored arrows point to black and white hair in (**b**). (**c**) Scatter plot of DoLP and albedo of different color hairs in the outdoor scene shows an inverse relationship between DoLP and albedo under skylight illumination. Error bars represent the standard deviation of the histogram for DoLP and albedo. The solid line is the least square fit to an equation of the Umov effect ($\Delta p=5040$ and $p=11689$).

Our study was conducted at noon when the sun was near its apex. We observed hair of varying colors under direct sunlight and skylight illumination, as shown in Fig. 5a. This figure displays the RGB radiance of various objects, including individuals with black, brown, and white hair. The DoLP of skylight changes with the sun’s position, with the highest DoLP observed 90° away from the sun. As indicated by magenta-colored arrows in Fig. 5b, the black hair of a standing person exhibited a high DoLP of around 0.39. In contrast, the white hair of a person sitting in a white shirt exhibited a low DoLP of approximately 0.05.

Figure 5c compares DoLP and albedo under partially polarized skylight and sunlight illumination obtained from hair samples of different individuals. The measured data follows the Umov effect’s inverse relationship between DoLP and albedo, similar to the indoor experiments depicted in Figs. 2, 3 and 4. The polarization properties of the scattered light obtained from the outdoor experiment are in good agreement with those obtained from indoor experiments.

### Single hair strand

An individual single hair strand was selected from each of the colored hair bundles. The single hair strands were mounted in parallel side by side, with adequate spacing, on a custom-made frame. The measurements were done using a Mueller matrix imaging polarimeter at two wavelengths, 450 nm and 589 nm, in a reflective mode. The polarimeter measures spatially dependent polarization properties of each single hair strand in the Mueller matrix^27^. Diattenuation, depolarization, and retardance can be calculated directly from the coefficients of the Mueller matrix.

**Figure 6:**
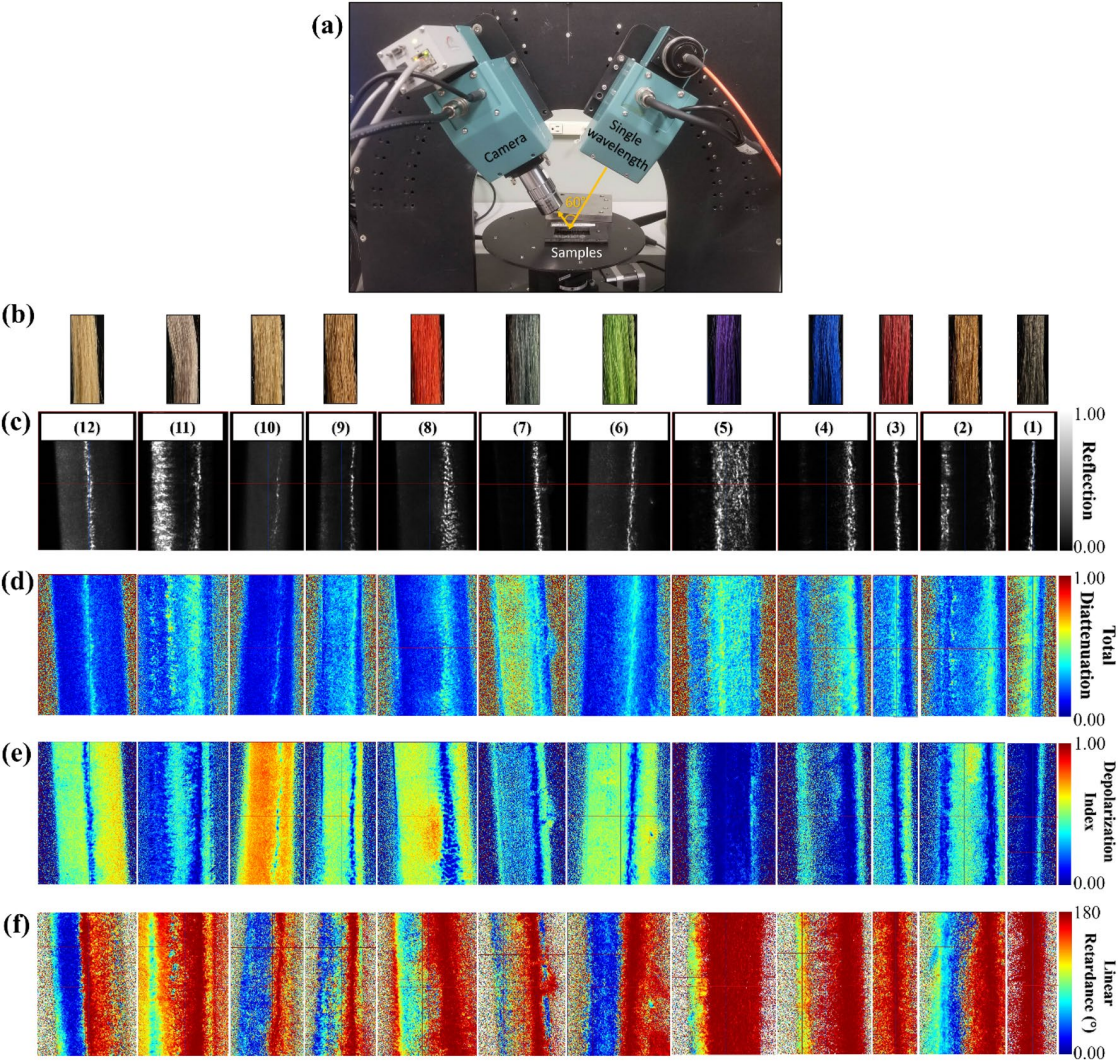
(**a**) Image shows the experimental configuration of the Mueller matrix imaging polarimeter in reflection mode with an incident angle at 60°, close to the Brewster angle. (**b**) Images are shown for the twelve different colored hair bundles: light yellow-white, gray-white, yellow-white, blond, red, dusty blue, green, purple, blue, dark red, brown, and black, from left to right. The single-hair strands are from these colored hair bundles. (**c**) Images of a single strand of hair of different colors are shown in reflection mode. All images were measured at a wavelength of 589 nm. (**d**) Images of the total diattenuation of a single strand of hair of different colors are shown. (**e**) Images of the depolarization index of a single strand of hair of different colors are shown. (**f**) Images of the linear retardance of a single strand of hair of different colors are shown.

In Fig. 6a, the experiment setup is shown, using an incident angle of $\theta ={60}^{\circ }$ close to the Brewster angle for the air-hair interface. Collimated light from a polarization state generator is directed at a single hair strand, which reflects at different angles due to the hair’s cylindrical shape. The illumination source has a wavelength of 589 nm, and a polarization state analyzer collects the reflected light. The colors of the hair strands correspond to those of the hair bundles shown in Fig. 6b. In Fig. 6c, the images of individual hair strands reveal a bright vertical band of reflected light attributed to specular reflection. These bands are spatially varying and nonuniform, indicating that the hair’s surface is not entirely smooth, and the cross-section of the hair is not perfectly circular.

First, the diattenuation of the single hair strands is examined. Diattenuation is an optical property of a material that measures the preferential absorption or scattering of light of one polarization compared with its orthogonal polarization, including linear, elliptical, and circular diattenuation. An ideal polarizer has a diattenuation of value one. Figure 6d shows the diattenuation maps of different colored single hair strands, where high diattenuation is observed in regions of the vertical reflection bands. The locations of high diattenuation regions are close to those with high specular reflections in the same hair, as shown in Fig. 6c. The high diattenuation indicates Fresnel reflection between the air/hair interface at or close to the Brewster angle.

Next, the depolarization of the single hair strands is analyzed, which measures the conversion of polarized light to unpolarized light. An ideal depolarizer has an index value of one. Figure 6e shows the depolarization index maps of the single hair strands, presenting vertical bands of high depolarization index in yellow, green, and red colors. The locations of these regions are close to those with little reflection, as shown in Fig. 6c. The high depolarization index in these regions indicates multiple reflections and scattering and a single reflection to a large angle, which the polarization state analyzer cannot collect. Figure 6f shows the color maps of linear retardance of the single hair strands, which is attributed to the inherent birefringence of the hair. Hair of lighter colors shows a small amount of linear retardance due to the light transmitted through the hair strand and reflected from the back surface of the hair^28^. Histograms of retardance of the single hair strands are included in the Supplementary Information.

Finally, measurements for the illumination wavelength of 450 nm were carried out on the same set of samples to study the wavelength dependency of the polarization properties of hair, with images of the data set provided in the Supplementary Information. Components of the Mueller matrix for each hair color at two wavelengths, 450 nm and 589 nm, are analyzed to determine the effect of illumination wavelength on polarization properties.

**Figure 7:**
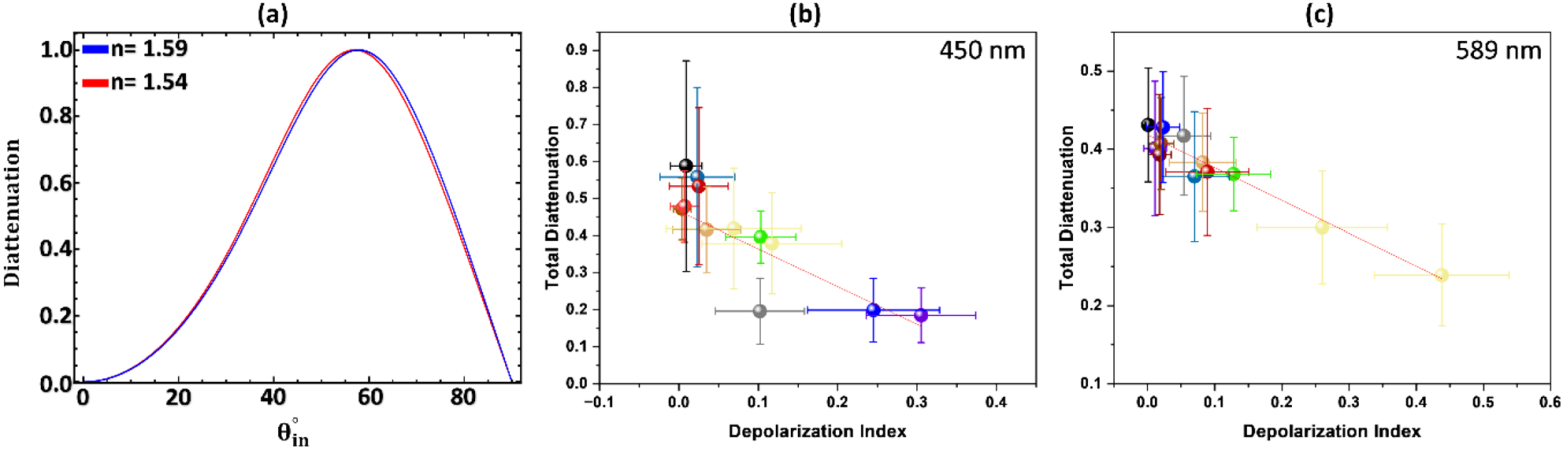
(**a**) Theoretical diattenuation curve is plotted as a function of incident angle for refractive indices 1.54 and 1.59. (**b**) Scatter plot shows the total diattenuation for twelve different single hair strands vs. depolarization index at a wavelength of 450 nm. The color of each point is chosen to match the color of the hair. The solid line shows a linear regression fit with R^2^ = 0.74. The error bars represent the standard deviation of the histogram for total diattenuation and depolarization. (**c**) Scatter plot shows the total diattenuation for twelve different single hair strands vs. depolarization index at a wavelength of 589 nm. The solid line shows a linear regression fit with R^2^ = 0.95. The error bars represent the standard deviation of the histogram for total diattenuation and depolarization.

The way hair responds to polarized light illumination is dependent on the wavelength. Keratin, the primary component of hair, has normal dispersion in the visible spectrum, but a change in the refractive index does not result in a significant change in diattenuation. Figure 7a demonstrates the diattenuation function of the incident angle at the air/hair interface for two refractive indices, 1.54 and 1.59, which correspond to the refractive index of keratin at 350 nm and 650 nm, respectively^29^. The total diattenuation and depolarization at the two wavelengths have a high correlation. Scatter plots in Fig. 7b,c indicate a general linear trend of decreasing total diattenuation with increasing depolarization index regardless of wavelength. This is attributed to the Umov effect, where the polarization of the incident light is scattered and scrambled, resulting in lower intensity and a lower degree of polarization of the reflected light. The spectral Umov effect is also observed where multiple scattering at a specific wavelength or color increases the albedo and decreases the polarization of the scattered light. This is supported by data from Mueller matrix imaging experiments, shown in Fig. 7b and c. In comparison, single black hair exhibits high values of total diattenuation, 0.57 and 0.43, at 450 nm and 589 nm, respectively, due to its large amount of melanin, which has a higher absorption at 450 nm than at 589 nm^30^.

**Figure 8:**
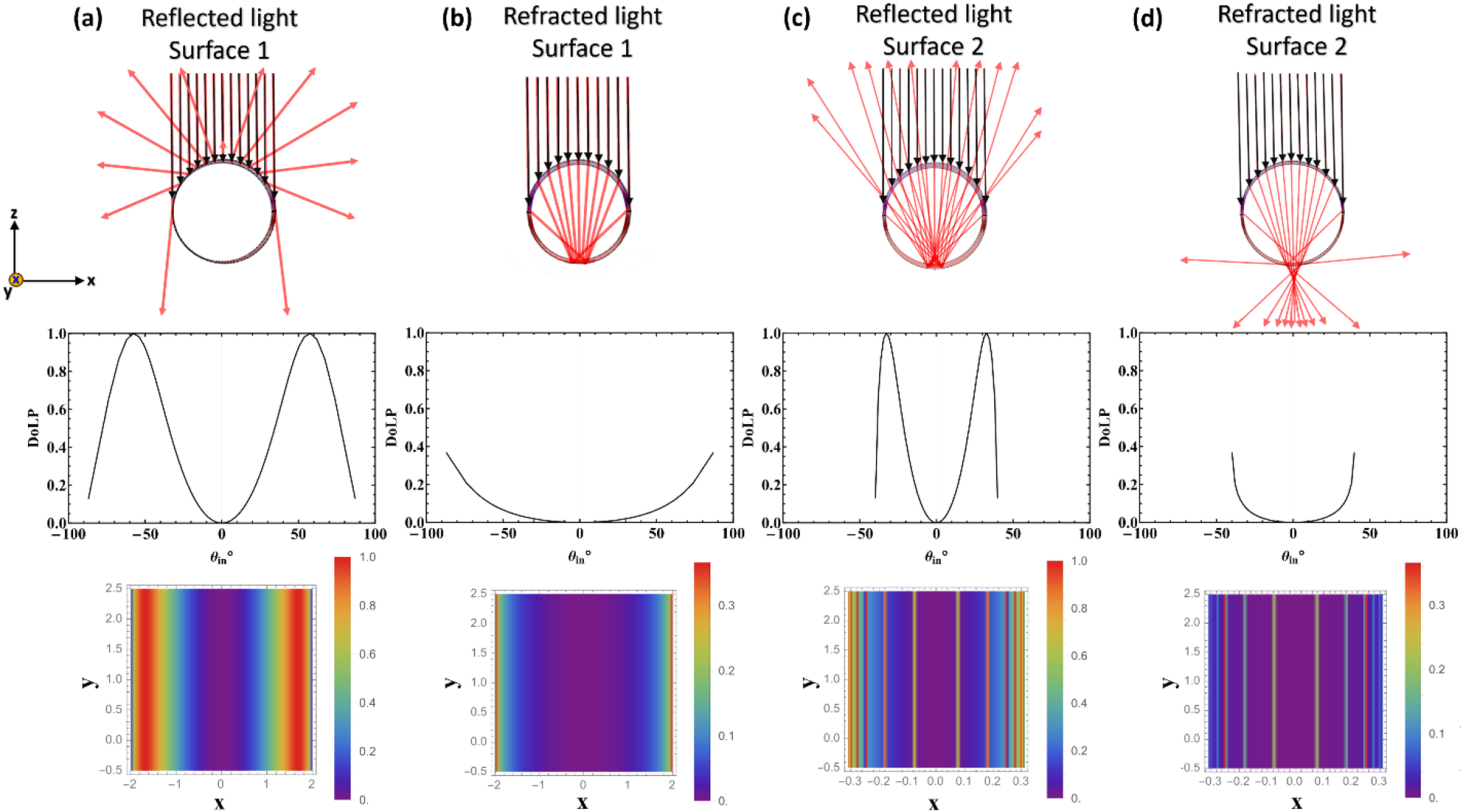
Results of polarization ray tracing calculations of hair are shown. The hair strand parallels to the y-axis, whereas the incident rays are along the z-axis. (**a**) The cross-section diagram shows the reflection of collimated rays by the front surface of the hair (top). DoLP of the different rays is plotted as a function of angle (middle). DoLP of the different rays is shown as a function of position along the hair (bottom). (**b**) Similar results are shown for transmitted or refracted rays for the front surface. (**c**) The transmitted rays from the front surface propagate inside the hair and are reflected from the back surface, i.e. the hair/air interface. (**d**) The transmitted rays from the front surface propagate inside the hair and are transmitted through the back surface.

High DoLP and diattenuation were observed in images of different types of hair. In order to understand the mechanism that generates the polarized light, we perform detailed polarization raytracing of a single hair strand using the commercial software Polaris-M (Airy Optics, Tucson, Arizona). The hair strand is modeled as a circular dielectric cylinder with a uniform refractive index of 1.56. The polarization properties of light rays after reflection and refraction through different air/hair interfaces are summarized in Fig. 8. Unpolarized incident light is collimated and propagated in parallel to the z-axis of the hair. Two important surfaces are the semi-circular front air/hair surface and the semi-circular back hair/air surface. Both the reflected and refracted rays for the two surfaces are calculated.

The top rows of Fig. 8 display the cross-section of the hair and light rays, while the last row exhibits the spatial distribution of DoLP for different surfaces and light rays. In Fig. 8a, the front surface’s reflected rays are linearly polarized at 57.3° and − 57.3° propagation angles, displayed as two vertical bands of unity DoLP. Figure 8b showcases refracted rays from the front surface, exhibiting a maximum DoLP of around 0.4 as the rays propagate through the cylindrical hair to the back surface. Figure 8c illustrates the reflected ray at the hair-air interface at the back surface, with rays linearly polarized at propagation angles of 32.7° and − 32.7°. These rays are only observable under low loss and scattering conditions. Figure 8d shows refracted rays from the back surface, with a maximum DoLP of around 0.4. Our analysis suggests that hair reflection from both front and back surfaces can convert unpolarized light to linearly polarized light. We assumed the incident light ray to have only a z component. However, additional results from polarization ray tracing for incident light rays with both z and y components are presented in the Supplementary Information. When the hair/air interface has total internal reflection (TIR) at a specific angle, linear polarized light can be transformed into elliptically polarized light. However, based on our experiments and calculations, elliptically polarized light is expected to be small since very few rays satisfy the TIR conditions.

## Discussion

This study focuses on understanding the polarized light interaction with human hair using polarization ray tracing calculations, full Stokes, and Mueller matrix imaging techniques. We examined the relationship between hair color, albedo, and the polarization state of light that reflects off hair. Polarization ray tracing calculations show the conversion of unpolarized light to polarized light by reflection and refraction of hair’s front and back surfaces.

Indoor and outdoor experiments revealed that the DoLP and albedo of hair exhibited an inverse relationship. Light-colored hair bundles exhibited high albedo and low DoLP. This can be attributed to their higher scattering and lower absorption properties. In contrast, dark-colored hair bundles showed low albedo and high DoLP due to increased absorption and reduced scattering. These observations support the Umov effect, which describes the inverse relationship between DoLP and albedo. The AoLP images provided insights into the specular and diffuse reflection. The AoLP for dark hairs has peak values close to 90°, indicating parallel alignment with the hair strands. This alignment suggests specular reflection near the Brewster angle, contributing to the high DoLP observed in dark hair bundles. The AoLP histogram for light hairs has two peak values, and the relative size of the peaks provides an estimate of the ratio between specular and diffuse reflections.

The spectral Umov effect can be used to describe how DoLP varies with albedo for different hair colors. The total diattenuation of a single hair strand decreases roughly linearly with increasing depolarization for different color hairs. Lower diattenuation is observed when colored hair is illuminated by the same color light. The magnitude of diattenuation is wavelength and angle-dependent and is larger for hair with higher absorption. Further investigations and correlations between hair color and Mueller matrix components at multiple wavelengths could provide additional insights into the wavelength-dependent behavior of hair polarization.

In summary, light scattering of hairs is generally polarization and wavelength-dependent. Our findings can have implications in various fields, such as computer graphics, medical diagnosis, laser hair removal, cosmetic science, and forensics.

### Supplementary Information

Supplementary Information.

## Data Availability

The datasets used and/or analyzed during the current study are available from the corresponding author on reasonable request.
