# Supplementary material for: Polarization properties and Umov effect of human hair
Source: Sci Rep. 2024 Jan 3;14:412. doi: 10.1038/s41598-023-50457-x (PMC10764807; doi:10.1038/s41598-023-50457-x)
Supplement: Supplementary file 1 — Supplementary Information. [file 41598_2023_50457_MOESM1_ESM.docx]

**Polarization Properties and Umov Effect of Human Hair**

Alaa Hamdoh^1 *^, Sawyer Miller^2^, Yufei Gao^2^, Yang Zou^3^, Matthew Smith^3^, Linan Jiang^2^, and Stanley Pau^1,2 *^

^1^ Electrical and Computer Engineering, University of Arizona, Tucson, Arizona 85721, USA.

^2^ James C. Wyant College of Optical Science, University of Arizona, Tucson, Arizona 85721, USA

^3^ Axometrics, Inc., Huntsville, Alabama 35806, USA

*Corresponding authors: [ahamdoh@arizona.edu](mailto:ahamdoh@arizona.edu); [spau@optics.arizona.edu](mailto:spau@optics.arizona.edu)

# Supplemental Discussion 1: Mueller Matrix Imaging at 450nm

^
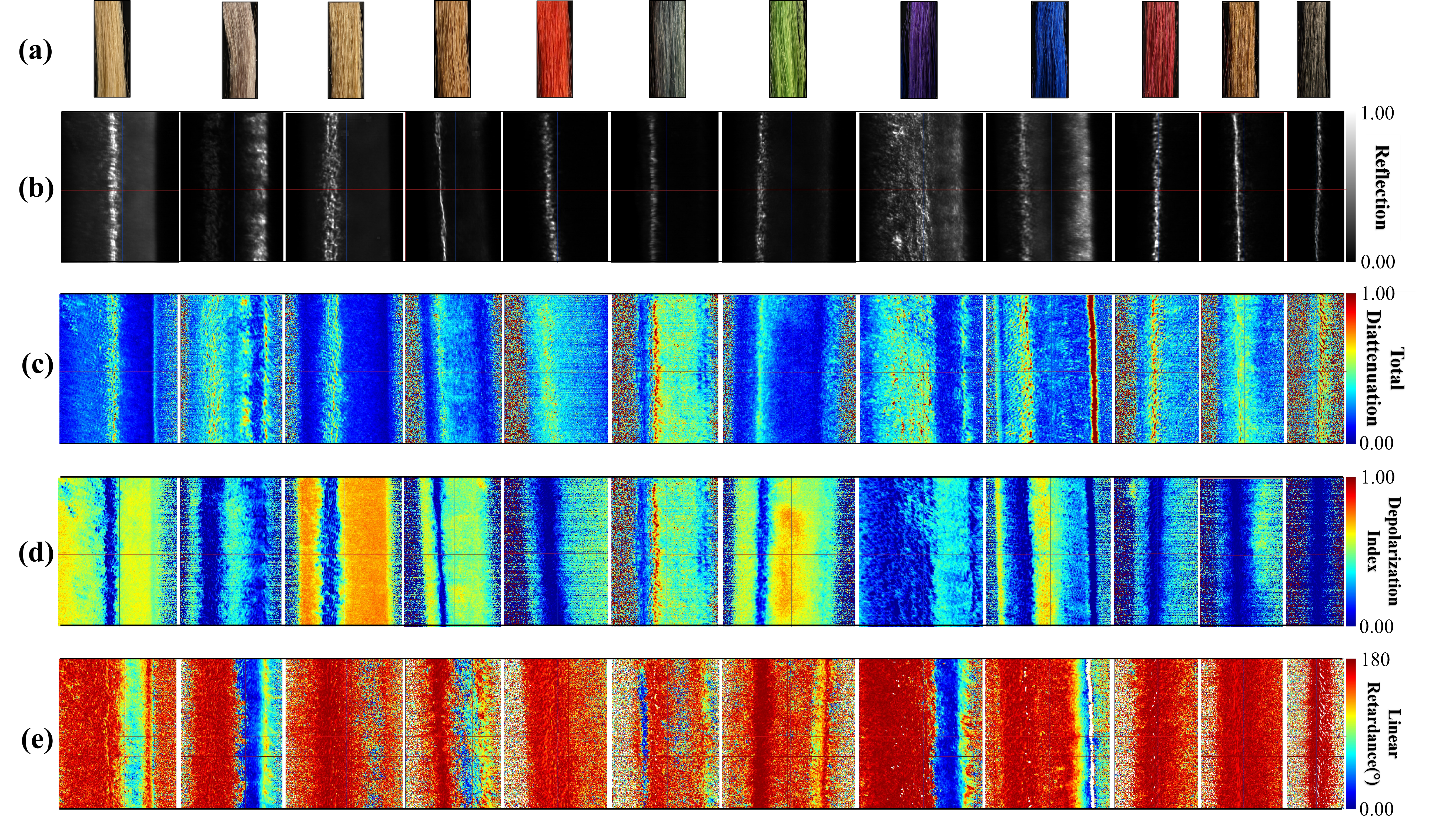
^

**Figure S1:** (a) Images are shown for the twelve different color hair bundles: light yellow-white, gray-white, yellow-white, blond, red, dusty blue, green, purple, blue, dark red, brown, and black, from left to right. (b) Images of a single strand of hair of different colors are shown in reflection mode. All images were measured at wavelength 450 nm. (c) Images of the total diattenuation of a single strand of hair of different colors are shown. (d) Images of the depolarization index of a single strand of hair of different colors are shown. (e) Images of the linear retardance of a single strand of hair of different colors are shown.

The Mueller matrix images of hair strands in different colors can offer valuable information on diattenuation, depolarization index, and linear retardance, particularly for 450 nm. To provide a comprehensive view, these discoveries are displayed in Figure S1 and can be contrasted with the details presented in Figure 6 for 589nm. Figure S1(d) reveals that areas with diffuse reflections in light and dark-colored hair, like blue and violet, have high depolarization caused by numerous reflections and scattering from the hair surfaces. This observation is further emphasized by a comparison with Figure 6(e).

# Supplemental Discussion 2: Histograms of Retardance from Mueller Matrix Imaging Experiments

Research has shown that hair exhibits birefringence, which can split light into two polarizations. This effect is observed across a range of wavelengths, including 450nm and 589nm, and is due to the intrinsic properties of hair. We can obtain a histogram of its linear retardance by measuring the Mueller matrix image of a single hair strand. Light-colored hair tends to have a wider range of retardance than darker hair, as more light can pass through lighter-colored hair. Additionally, retardance distribution varies depending on the wavelength of light used. For instance, white hair at 589nm typically exhibits two peaks of retardance at 20° and 180°, while at 450nm, it shows retardance from 90° to 180°. Purple hair at 589nm peaks at 180°, while at 450nm, it has a secondary peak at 20° and a major peak at 180° due to reflection from the front surface of the hair, as shown in Figure 8(c). The exact reasons for these variations in retardance distribution are still being investigated and depend on factors like the hair's absorption, birefringence distribution, and depolarization.


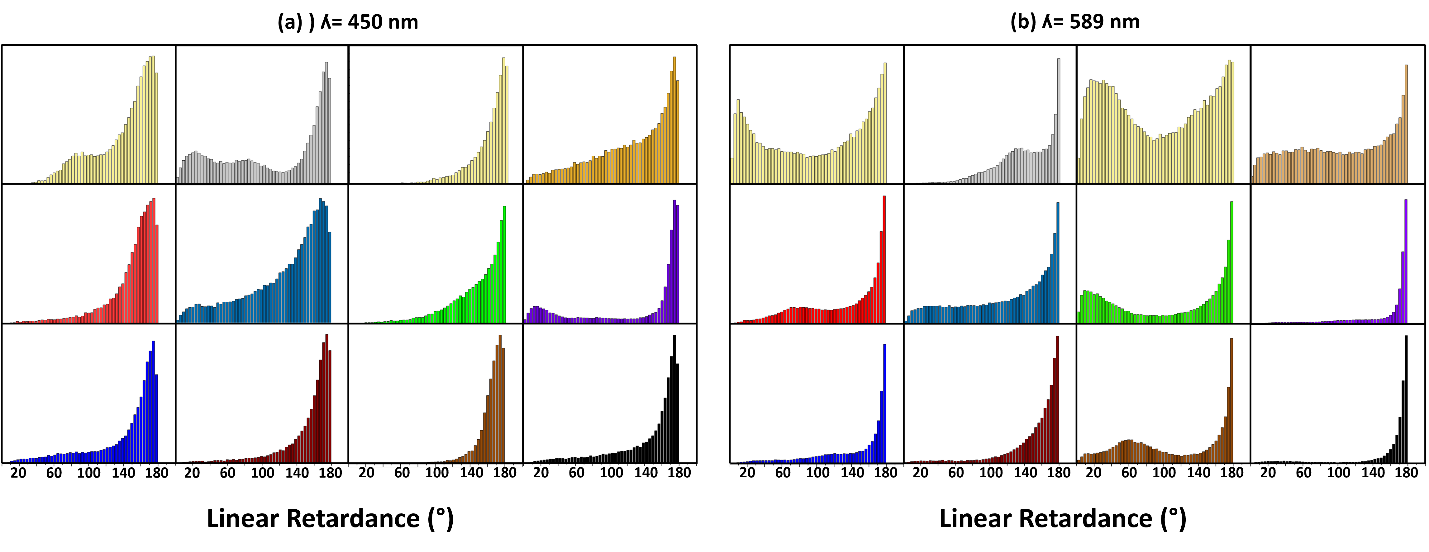


**Figure S2:** Histograms of linear retardance for different hair colors are shown. In the top row, the hair bundle colors are light yellow-white, gray-white, yellow-white, and blond, from left to right. The middle row has red, dusty blue, green, and purple hair bundle colors from left to right. The bottom row has blue, dark red, brown, and black hair bundle colors from left to right. (a) Linear retardance histograms of individual hair strands were taken with 450 nm illumination. (b) Linear retardance histograms of individual hair strands were taken with 589 nm illumination.

# Supplemental Discussion 3: Polarization Ray Tracing Study of Total Internal Reflection of Hair


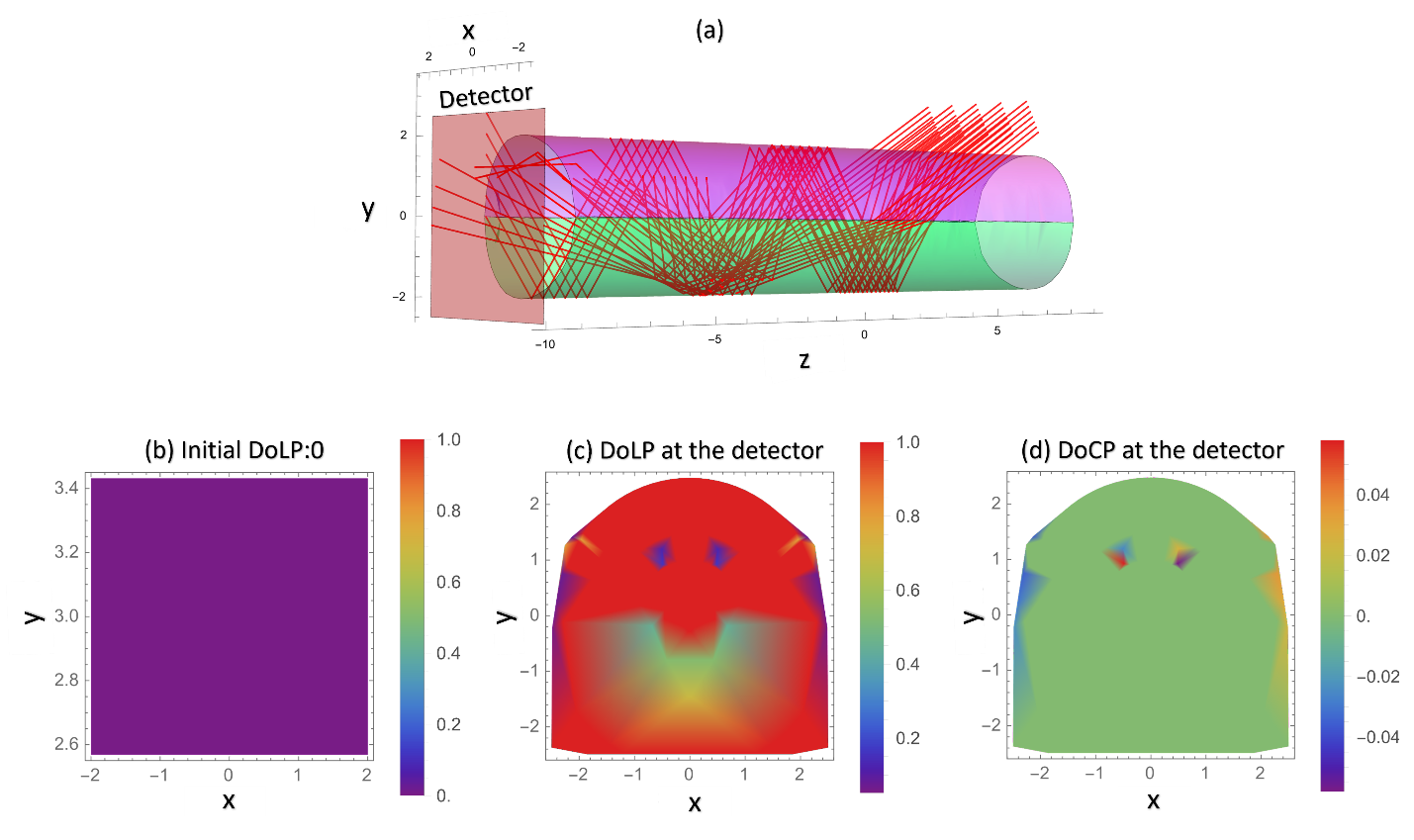


**Figure S3:** Polarization ray tracing calculation is performed for a single strand of hair. The hair strand is parallel to the z-axis. (a) The cross-section diagram shows the polarization states of different light rays. (b) The DoLP of initially collimated rays is zero, i.e., the input rays are unpolarized. (c) The DoLP map of different rays is shown at the detector after propagation through the cylinder. (d) The detector shows a DoCP map of different rays after propagation through the cylinder.

We used polarization ray tracing to analyze the degree of linear polarization (DoLP) and elliptical/circular polarization (DoCP) after passing through a single strand of hair. Our model assumed the hair to be a cylinder with a uniform refractive index of 1.56 and non-depolarization properties. The results indicate that light rays reflect multiple times within the cylinder through total internal reflection and then exit at one end (Figure S3(a)). In actual hair, scattering centers within the hair can cause light leakage along the hair. The input collimated rays, unpolarized light, result in a DoLP of zero, as shown in Figure S3(b). The DoLP map of different rays at one end of the cylinder after propagation through the cylinder is presented in Figure S3(c). Red indicates areas of high DoLP where the reflected angle is close to the Brewster angle. Figure S3(d) shows zero DoCP in most of the area, as some rays did not meet the conditions for total internal reflection. However, a small amount of elliptically polarized light is displayed at two locations due to a phase shift from total internal reflection. These results align with our experiment's outcomes in Figure 4(b).
